# Comparative Surfaceome Analysis of Clonal *Histomonas meleagridis* Strains with Different Pathogenicity Reveals Strain-Dependent Profiles

**DOI:** 10.3390/microorganisms10101884

**Published:** 2022-09-21

**Authors:** Marcelo de Jesus Ramires, Karin Hummel, Tamas Hatfaludi, Petra Riedl, Michael Hess, Ivana Bilic

**Affiliations:** 1 Clinic for Poultry and Fish Medicine, Department for Farm Animals and Veterinary Public Health, University of Veterinary Medicine Vienna, Veterinärplatz 1, A-1210 Vienna, Austria; 2 VetCore Facility for Research, University of Veterinary Medicine Vienna, Veterinärplatz 1, A-1210 Vienna, Austria; 3 Christian Doppler Laboratory for Innovative Poultry Vaccines (IPOV), University of Veterinary Medicine Vienna, Veterinärplatz 1, A-1210 Vienna, Austria

**Keywords:** *Histomonas meleagridis*, surface proteome, virulence factors, attenuation, LC-MS/MS, OMICs, host–parasite interaction, intestinal protozoan

## Abstract

*Histomonas meleagridis,* a poultry-specific intestinal protozoan parasite, is histomonosis’s etiological agent. Since treatment or prophylaxis options are no longer available in various countries, histomonosis can lead to significant production losses in chickens and mortality in turkeys. The surfaceome of microbial pathogens is a crucial component of host–pathogen interactions. Recent proteome and exoproteome studies on *H. meleagridis* produced molecular data associated with virulence and *in vitro* attenuation, yet the information on proteins exposed on the cell surface is currently unknown. Thus, in the present study, we identified 1485 proteins and quantified 22 and 45 upregulated proteins in the virulent and attenuated strains, respectively, by applying cell surface biotinylation in association with high-throughput proteomic analysis. The virulent strain displayed upregulated proteins that could be linked to putative virulence factors involved in the colonization and establishment of infection, with the upregulation of two candidates being confirmed by expression analysis. In the attenuated strain, structural, transport and energy production proteins were upregulated, supporting the protozoan’s adaptation to the *in vitro* environment. These results provide a better understanding of the surface molecules involved in the pathogenesis of histomonosis, while highlighting the pathogen’s *in vitro* adaptation processes.

## 1. Introduction

*Histomonas meleagridis* is an extracellular parasitic protozoan of the order Tritrichomonadida [1] and the causative agent of histomonosis (syn. Blackhead disease) in gallinaceous birds [2].

Histomonosis can cause high mortality in turkeys, leading to casualties of up to 100%. In chickens, the disease is less severe, displaying a reduction in egg production. Nevertheless, it is often diagnosed in laying and breeder hens, where a considerable increase in mortality can be observed, leading ultimately to substantial economic losses. [3,4]. For decades, histomonosis was well controlled with antihistomonal products used for therapy and prophylaxis [5]. As a result, research on the parasite came to a halt. In the last two decades, new drug legislation in the European Union and USA banned all available treatment methods for food-producing animals [5]. This, combined with the increasing popularity of free-range farming, led to a substantial increase in *H. meleagridis* outbreaks in poultry flocks [6]. Currently, only a prototype live vaccine based on an *in vitro* attenuated strain has been shown to prevent damage caused by histomonosis [6].

As a direct consequence of the re-emergence of this “old” pathogen, investigations on its molecular biology have gained new strength. Most of the initial molecular investigations focused on the phylogeny of *H. meleagridis*, and just recently the “omics”-based research has started [7]. Proteome and exoproteome studies identified differentially expressed proteins in virulent and attenuated strains using mass spectrometry combined with gel-based and gel-free methods, supported by sequences from a transcriptome database [8,9,10,11,12]. In addition, the recently reported full genome sequences of a virulent *H. meleagridis* strain and an attenuated *H. meleagridis* strain now provide the underlying genomic data [13].

Yet, the molecular understanding of the *H. meleagridis* surface proteome is still very limited, although surface-associated proteins are at the forefront in host–pathogen interactions. Their possible roles include: adherence to the mucosal tissue; virulence; transport; resistance to environmental conditions; and, overall, long-term survival of the pathogen [14,15].

Exploiting the high affinity of the biotin–avidin bond by cell surface biotinylation has become one of the most favorable methods for extracting surface proteins. It produces the lowest rates of contamination with cytosolic proteins when compared to other methods, such as trypsin shaving and cell fractionation [16].

The molecular studies on *H. meleagridis* were built upon its *in vitro* propagation, which is based on a monoxenic clonal culture, enabling a well-defined platform for precise and thorough molecular analyses [17,18].

Here we aimed to identify and characterize the proteins associated with the surface of *H. meleagridis.* Alongside high-throughput proteomic analysis, we present a description of its surface proteome (surfaceome), with a special focus on the differences in protein regulation between a virulent strain and an attenuated strain originating from the same cell.

## 2. Materials and Methods

### 2.1. Protozoan Cultures

All biotinylation experiments were performed using virulent and attenuated, monoxenic mono-eukaryotic *H. meleagridis* cultures propagated *in vitro*, *H. meleagridis* turkey/Austria/2922-C6/04-10x/16x-DH5α and *H. meleagridis* turkey/Austria/2922-C6/04-290x/48x-DH5α, respectively [18]. Strain labeling adhered to the following rules: host/country of isolation/protocol number–clone number/year of isolation–number of passages in xenic conditions/number of passages in monoxenic conditions–bacterial strain in monoxenic conditions. The histomonads were co-cultivated with the bacterial strain *E. coli* DH5α as a supplement. The cultures were incubated at 40 °C in 28 mL of RPMI Media 1640 (Gibco, Invitrogen, Lofer, Austria) with 15% heat-inactivated fetal bovine serum (FBS) (Gibco, Invitrogen, Lofer, Austria) and 0.25% sterilized rice starch (Carl Roth GmbH + Co. KG, Karlsruhe, Germany). Cells were passaged every 48 h.

### 2.2. Biotinylation of Surface-Associated Proteins

**Figure 1:**
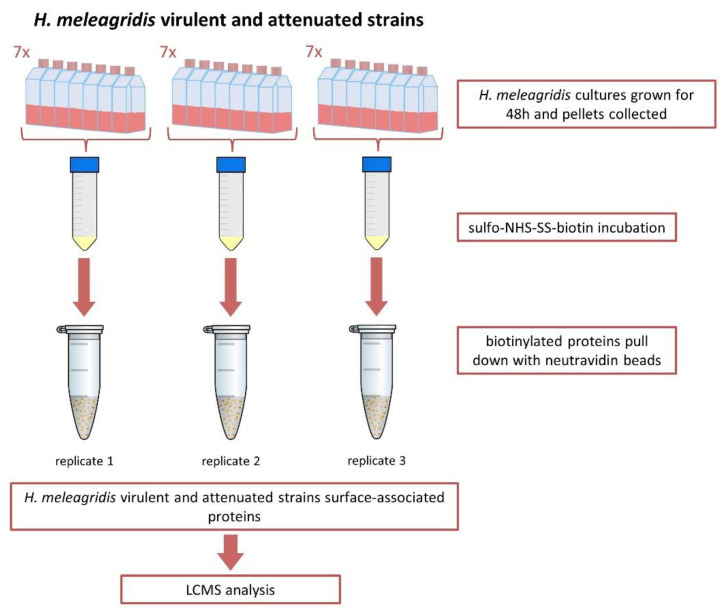
Experimental set-up. Schematic representation of the experimental set-up used for biotinylation and purification of *H. meleagridis* surface-associated proteins. The protozoan was incubated in seven T75 flasks. After 48 h, the cultures were centrifuged, and the pellets were collected and incubated with sulfo-NHS-SS-biotin. Biotinylated proteins were pulled down with neutravidin beads, selecting for surface-associated proteins. Samples were analyzed with mass spectrometry.

*Histomonas meleagridis* cultures were set up in 600 mL of RPMI growth media divided into 7x T75 flasks and incubated at 40 °C for 48 h. To ensure the reproducibility of the protocol, three technical replicates were prepared for each strain. Each technical replicate comprised 7 biological replicates grown in parallel in T75 culture flasks (Figure 1).

When complete, cells were transferred into 50 mL falcon tubes (Sarstedt, Wiener Neudorf, Austria) and centrifuged at 200× *g* for 5 min at room temperature. The supernatant was discarded, and the pellets were combined. The pelleted parasites ranging in numbers between 1 and 5 × 10^8^ cells were re-suspended with 0.5 mg/mL EZ-Link sulfo-NHS-SS-biotin (Pierce, Thermo Fisher Scientific, Vienna, Austria) in 50 mL of prewarmed PBS and incubated for 30 min at 40 °C (Figure 1). Upon completion, the biotinylation reaction was quenched by the addition of 50 mM Tris-HCl, pH 7.5. When complete, cells were centrifuged at 200× *g* for 5 min at room temperature. To ensure the removal of *E. coli* DH5α from the sample, whilst maintaining the histomonad within the pellet, the cells were washed four times with 25 mL of prewarmed RPMI media before proceeding to protein extraction.

### 2.3. Assessing Membrane Permeabilization after Biotinylation

Cell lysis during biotinylation was assessed by counting live cells before and after the biotinylation procedure. Cell numbers were assessed using trypan blue (Gibco, Invitrogen, Lofer, Austria) and a Neubauer hemocytometer (Sigma-Aldrich, Vienna, Austria).

Only samples with cell lysis below 10% after biotinylation were used for further analyses.

### 2.4. Membrane Protein Enrichment and Purification of Biotinylated Proteins

Biotinylated cells were re-suspended in Triton X-100 lysis buffer (50 mM Tris/HCl (pH7.4), 150 mM NaCl, 1 mM EDTA, 1% Triton X-100). Due to the complex nature of the sample, and to ensure cell lysis, samples were placed in 2 mL Eppendorf tubes and homogenized twice for 2 min at 28 Hz using TissueLyzer (Qiagen, Hilden, Germany). The cell lysate was centrifuged at 10,000× *g* for 2 min at 4 °C, and the supernatant was collected. Membrane and membrane-associated proteins were enriched by ultracentrifugation at 100,000× *g* for 1 h and 45 min at 4 °C and re-suspended in buffer (20 mM HEPES (pH 7.4), 10 mM KCl, 2 mM MgCl_2_, 1 mM EDTA, 1 mM EGTA). Prior to use in the pull-down assay, NeutrAvidin-Sepharose beads (Pierce, Thermo Scientific, Vienna, Austria) were equilibrated over two washes with 500 μL PBS. Biotinylated proteins were bound onto the neutravidin-coated beads during a one-hour incubation at room temperature on an end-over-end rotator. The beads were then washed three times with 500 μL of a PBS and protease inhibitor (Merck, Austria, Vienna, Austria) solution. Biotinylated proteins were eluted using CHAPS-DTT lysis buffer (150 mM KCl, 50 mM HEPES, 0.1% CHAPS, 50 mM DTT) in a one-hour incubation at room temperature on an end-over-end rotator (Figure 1).

To control for unspecific binding of NeutrAvidin-Sepharose, a non-biotinylated technical replicate was prepared. Eluted proteins from biotinylated and control non-biotinylated samples were analyzed on a silver-stained SDS-PAGE.

### 2.5. One-Dimensional SDS-PAGE (Sodium Dodecyl Sulfate–Polyacrylamide Gel Electrophoresis)

*Histomonas meleagridis* biotinylated protein’s electrophoretic profile was analyzed by 1D SDS-PAGE. From each preparation, 20 μL of cell lysate was separated on 8% SDS-PAGE for 90 min with constant 120 V. Separated proteins were visualized using the silver-staining protocol [19].

### 2.6. Sample Preparation and nanoHPLC-Orbitrap MS/MS Analysis

Protein extracts were digested applying a filter-aided sample preparation protocol based on the work of Wisniewski et al. (2009) and Wisniewski (2016) with adaptations for the use of Trypsin/Lys-C mix (Promega Technical Manual) [20,21]. In brief, Pall Nanosep centrifugal devices with Omega membrane and a cut-off of 10 kDa were washed with 8 M urea in 50 mM Tris (pH 8.0): 500 µL/500 µL/300 µL followed by centrifugation between each step (10,000× *g* for 15 to 20 min). Thirty micrograms of protein were diluted with 8 M urea in 50 mM Tris (pH 8.0) to a total volume of 500 µL and loaded onto the filter before centrifugation. A reduction in 20 mM aqueous dithiothreitol for 30 min at 37 °C on a thermomixer was followed by alkylation in 60 mM aqueous iodoacetamide for 30 min at 25 °C on the filter. After two washing steps with 100 µL of 50 mM Tris, proteins were digested with Trypsin/Lys-C mix (Promega, Vienna, Austria) for 14 h overnight at 37 °C. Peptides were extracted in three steps each of 50 µL 50 mM Tris with subsequent centrifugation. Peptides were acidified with trifluoroacetic acid to a pH below 2.

Peptide clean-up was achieved with C18 spin columns (Pierce Thermo Fisher, Vienna, Austria) according to the manufacturer’s instructions before peptide analysis using nanoRSLC-ESI-Orbitrap MS/MS [22]. Three technical replicates were injected and analyzed per biological replicate.

### 2.7. H. meleagridis Proteome Database

The *H. meleagridis* proteome database was derived by conceptual translation of coding genomic sequences from virulent and attenuated *H. meleagridis* strains [13]. To ensure uniformity and the full coverage of the annotated protein-coding sequences, both datasets, virulent and attenuated, were merged. In the final proteome database, duplicate protein-coding sequences were removed, and one copy was retained under its initial accession number. Proteins for which the coding sequence was present in only one genomic dataset (virulent or attenuated) remained in the proteome database under their initial accession number. Identical proteins with different accession numbers were kept in the final proteome dataset.

### 2.8. Identification and Quantification of Surface-Associated Proteins

Evaluation of raw data was accomplished with Proteome Discoverer 2.4 (Thermo Fisher Scientific, Vienna, Austria). A combination of the *H. meleagridis* proteome database described above, the UniProt database for *E. coli* (taxonomy 83333, www.uniprot.org, accessed on 25 June 2019) and a common contaminant database (https://www.thegpm.org/crap/, accessed on 25 June 2019) was used. The following search parameters were applied: trypsin as an enzyme; maximally 2 missed cleavages; 10 ppm precursor mass tolerance and 0.02 Da fragment mass tolerance; dynamic modifications allowed were oxidation/+15.995 Da (M)/Biotin:Thermo-21328/+389.090 Da (K)/CAMthiopropanoyl/+145.020 Da (K), N-terminal modifications Biotin:Thermo-21328/+389.090 Da/Acetyl/+42.011 Da/CAMthiopropanoyl/+145.020 Da and static modification Carbamidomethyl/+57.021 Da (C).

For intensity-based label-free quantification (LFQ), resulting protein abundance raw values were exported for further analysis with the DEP package in R [23]. Prior to the import into R, *E. coli* proteins and the remaining proteins with more than two missing values per strain were excluded from the quantification analysis, which used all nine technical/biological replicates per strain. Proteins detected in only one strain (“ON/OFF proteins”) were included if values in all 9 technical/biological replicates were available from that strain whilst the values for the other strain were missing. Afterward, the technical replicates were aggregated by the mean. Statistical analysis of the virulent vs. the attenuated strain by *t*-test was performed according to the DEP script including the normalization of protein abundances and imputation of missing values by zero. From these, proteins recognized with more than two tryptic peptides and displaying a fold change higher than 2-fold with an adjusted *p*-value lower than 0.05 were considered to be upregulated in our analysis.

### 2.9. Re-Analysis of H. meleagridis Proteome and Exoproteome Data

Raw data of previously published experiments [10,12] were re-analyzed with the appropriate software packages for SWATH data: ProteinPilot Software 5.0.2, Sciex (Framingham, USA), PeakView 2.2, Sciex (Framingham, USA), and MarkerView, 1.3.1.1, Sciex (Framingham, USA), as stated in the original publications using the combination of the new *H. meleagridis* proteome database, the UniProt database for *E. coli* (taxonomy 83333, www.uniprot.org, accessed on 25 June 2019) and a common contaminant database (https://www.thegpm.org/crap/, accessed on 25 June 2019) as described above. Exported abundance values were used for further statistical evaluation with the DEP package in R as mentioned above.

### 2.10. In Silico Analysis

For the identification of secretion signals, unconventional secretion and transmembrane domains, the following programs were used with their default settings: **SignalP 4.1 Server**, (https://services.healthtech.dtu.dk/service.php?SignalP-4.1, accessed on 4 May 2021), **SecretomeP 2.0 Server** (https://services.healthtech.dtu.dk/service.php?SecretomeP-2.0, accessed on 4 May 2021) and **TMHMM 2.0 Server** (https://services.healthtech.dtu.dk/service.php?TMHMM-2.0, accessed on 4 May 2021).

### 2.11. RNA Extraction and Quantitative Reverse Transcriptase Polymerase Chain Reaction (RT-qPCR) Analysis

To confirm the upregulation, four genes upregulated in the virulent strain, namely alpha-amylase, clan CD family C13 asparaginyl endopeptidase-like cysteine peptidase, LysM domain-containing protein and surfactant B, were selected and analyzed by quantitative reverse transcription real-time polymerase chain reaction (RT-qPCR). For that purpose, *H. meleagridis* virulent (*H. meleagridis* turkey/Austria/2922-C6/04-10x/18x-DH5α) and attenuated (*H. meleagridis* turkey/Austria/2922-C6/04-290x/52x-DH5α) cultures were grown in RPMI medium 1640 (Gibco, Invitrogen, Lofer, Austria) containing sterilized rice starch (0.25%) (Carl Roth GmbH + Co. KG, Karlsruhe, Germany) and 15% heat-inactivated fetal bovine serum (FBS) (Gibco, Invitrogen, Lofer, Austria) for 6 and 48 h. Upon reaching the collection time point, the samples were centrifuged at 200× *g* for 5 min at room temperature and *E. coli* DH5α was removed over 4 washing steps, carried out in the same fashion as the biotinylation protocol. The final supernatant was discarded, and the pellets were re-suspended in a 1:1 RNA-later and RNase-free water solution. The suspension was stored at −80 °C until further use. Total RNA was extracted from ~1.0 × 10^7^ cells/mL using the Direct-zol RNA MiniPrep Plus kit (Zymo Research Europe, Freiburg, Germany) following the manufacturer’s instructions and stored at −80 °C until use. Total RNA samples were pretreated with an RNase-Free DNase Set (Qiagen, Hilden, Germany) according to the manufacturer’s instructions to remove contaminating genomic DNA.

RNA quantity and quality were assessed using Qubit RNA High Sensitivity (HS) (Invitrogen, Lofer, Austria), NanoDrop 2000 (Thermo Fisher Scientific, Vienna, Austria) and Agilent 2100 Bioanalyzer System using Bioanalyzer High Sensitivity RNA Analysis kit (Agilent technologies, Vienna, Austria).

All RNA samples used in the present work showed a value for the 260/280 ratio ranging between 1.6 and 2.0. Ratio measurements for the 260/230 values were consistently between 2.0 and 2.3 when measured with NanoDrop 2000 (ThermoFisher Scientific, Vienna, Austria). Each RNA sample’s integrity (RIN) was assessed. RIN values for all samples ranged between 8 and 10.

Primers and probes were designed using the Eurofins Genomics qPCR Primer & Probe Design software (Eurofins, Ebersberg, Germany, https://eurofinsgenomics.eu/de/ecom/tools/qpcr-assay-design/, accessed on 4 May 2021) with default settings (Supplementary Table S1). The RT-qPCR was conducted using TaqMan chemistry alongside the Brilliant III Ultra-Fast QRT-PCR Master Mix kit (Agilent Technologies, Vienna, Austria). Primer concentrations ranging from 200 to 500 nM and probe concentrations ranging from 100 to 200 nM were tested with 10-fold serial dilutions of *H. meleagridis* DNA (100, 10, 1, 0.1, 0.01, 0.001 ng). The amplification and quantification of the selected group of genes was performed using the AriaMx real-time PCR system (Agilent Technologies, Vienna, Austria) with the Agilent AriaMx1.71 software (Version: 1.7.1902.1242, Agilent Technologies, Vienna, Austria). The thermal profile of real-time reactions was as follows: 1 cycle of reverse transcription at 50 °C for 10 min, 95 °C for 3 min, 40 cycles of amplification at 95 °C for 5 s and 60 °C for 10 s.

The optimal primer and probe concentrations with respective PCR efficiency values are listed in Supplementary Table S1.

The suitability of the Fe-hydrogenase target as a reference gene was tested with RNA samples prior to the analysis of other targets (Supplementary Table S1). The virulent and attenuated *H. meleagridis* samples were analyzed in duplicate, together with non-RT (non–reverse transcriptase) and NTC (non-template control) controls in order to assess for possible genomic DNA and overall PCR contamination. The mean CT value of each duplicate was used for gene expression analysis.

To account for the variation in sampling and RNA preparation, the CT values for all genes were normalized using CT values of the reference gene Fe-hydrogenase. To evaluate the results, all the values were given as fold change by using the 2^−∆∆CT^ formula [24]. In this formula, ΔCT was calculated for each strain separately, where ΔCT = CT (a target gene) − CT (a reference gene), followed by ΔΔCT = ΔCT (attenuated strain) − ΔCT (virulent strain) and finally 2^−∆∆CT^ to obtain fold change values.

Altogether, our RT-qPCR investigations were compliant with the MIQE guidelines [25].

## 3. Results

### 3.1. Selective Biotinylation of Surface-Associated Proteins

Cultures with live *H. meleagridis* were labeled with sulfo-NHS-SS-biotin to isolate its surface-associated proteins. All biotinylation experiments were performed at room temperature due to the protozoan sensibility to incubation at +4 °C. Empirical research has shown that *H. meleagridis* cell deterioration is manifested by the protozoan’s membrane fragmentation. As such, dead cells tend to lyse and disintegrate, and hence microscopic observations do not allow the detection of a permeabilized membrane. Thus, cell lysis during the biotinylation process was considered in assessing the possible contamination with cytosolic proteins. Cell numbers before and after biotinylation were determined using trypan blue with cell loss values always below 10%.

**Figure 2:**
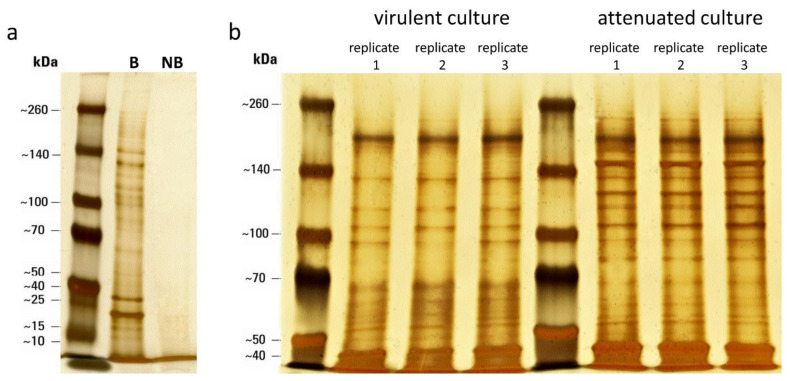
Biotinylation of surface-associated proteins. Proteins were separated on 8% SDS-PAGE gel and visualized by silver staining. (**a**) Results of pull-down assay using biotinylated [B] and non-biotinylated [NB] samples of *H. meleagridis*. (**b**) Surface-associated proteins isolated from 3 technical replicates of *H. meleagridis* virulent and attenuated strains.

Results of the pull-down assay using biotinylated and non-biotinylated samples demonstrated the specific binding of neutravidin-conjugated beads to biotinylated proteins (Figure 2a), which was confirmed by LCMS analysis of negative control (NB). Surface proteins from all three technical replicates of each strain displayed a very similar electrophoretic profile, whereas clear differences in the pattern of protein bands between the two strains were evident (Figure 2b).

### 3.2. Identification and Quantification of Surface-Associated Proteins

**Figure 3:**
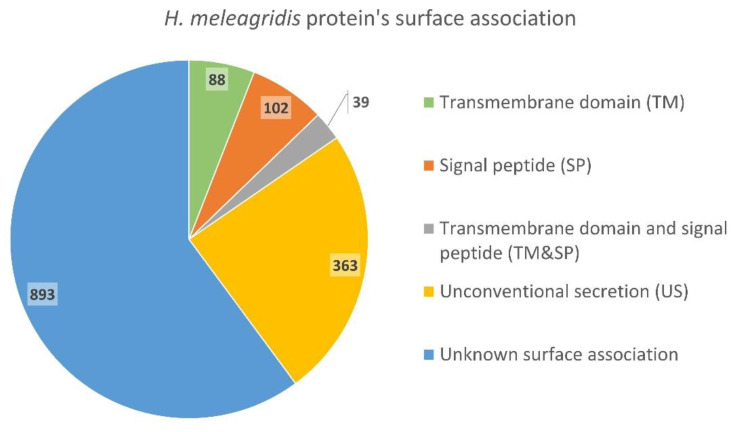
In silico prediction of surface and membrane association for *H. meleagridis* proteins identified by surface biotinylation.

Identification and quantification of proteins in the surface-enriched samples from the virulent and attenuated strains was achieved by liquid chromatography–mass spectroscopy (LCMS) investigation. Identification of putative surface-associated proteins in *H. meleagridis* revealed a total of 1485 proteins among the samples. From these, only 88 (5.9%) were predicted to contain one or more transmembrane domains (predicted with TMHMM software), 102 (6.9%) to contain a predicted signal peptide (predicted with SignalP software) in the N-terminal region, and 39 (2.6%) to have both a transmembrane domain and a signal peptide. Analysis with the SecretomeP software revealed 363 (24.4%) proteins predicted to be unconventionally secreted to the extracellular milieu, leaving the remaining 893 (60.2%) proteins without a clear correlation to the *H. meleagridis* surface (Figure 3 and Supplementary Table S2).

**Figure 4:**
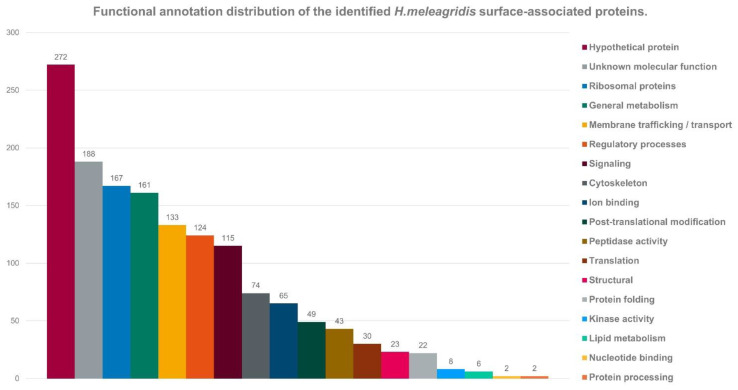
Distribution of functional annotations of the identified *H. meleagridis* surface-associated proteins. Functional groups were classified by BLAST homology analysis.

Using BLAST analysis, the identified putative surface-associated proteins were sorted into functional groups based on their annotation (Figure 4). The largest group comprised hypothetical proteins (18.3%, *n* = 272) and was closely followed by the group of ribosomal proteins (11.2%, *n* = 167). Proteins involved in general metabolic processes constituted 10.8% (*n* = 161) of the total dataset; additionally, 9% (*n* = 133) were found to be involved in membrane trafficking and transport, 8.3% (*n* = 124) were related to the protozoan regulatory processes, 7.7% (*n* = 115) were found to be small GTPases and 5% (*n* = 74) were found to be related to *H. meleagridis* cytoskeleton components (Figure 4).

**Figure 5:**
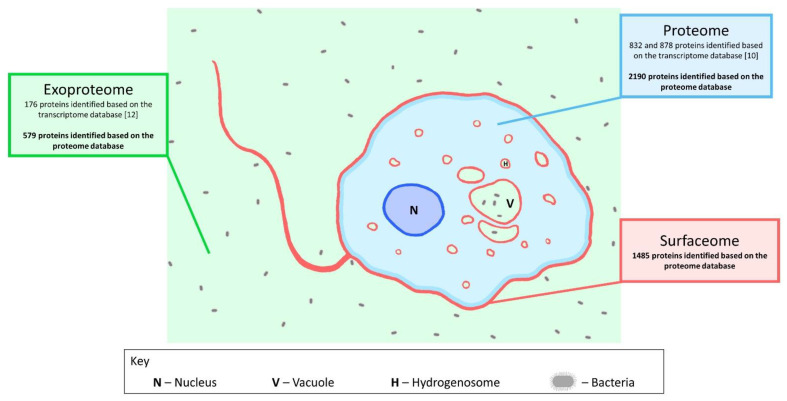
Schematic representation of the major *H. meleagridis* proteomic analyses to date.

To compare the surfaceome data with the already available data from proteome and exoproteome studies, we have re-analyzed the available shotgun LC-MS/MS measurement datasets using the new proteome database established from the recently published *H. meleagridis* genome [10,12,13] (Figure 5). A new analysis of the proteome LC-MS/MS measurements identified a total of 2189 proteins, significantly more than the 832 and 878 proteins previously identified for the attenuated and virulent strains, respectively [10] (Supplementary Table S3). A comparison with the surfaceome data identified 920 proteins present in both datasets (Supplementary Table S2). For the exoproteome, new data now comprise 579 proteins as opposed to the 176 proteins previously identified with the analysis using the proteome database [12] (Supplementary Table S4). In relation to the surfaceome, 233 proteins were found to be present in both exoproteome and surfaceome analyses (Supplementary Table S2).

The quantitative analysis of the surfaceome data identified a total of 67 proteins to be, significantly, differentially expressed (≥2-fold and *p*-value < 0.05). In the virulent strain, 22 proteins were upregulated, as opposed to 45 upregulated proteins in the attenuated strain (Table 1 and Table 2). Remarkably, 9 out of the 22 upregulated proteins in the virulent and 10 out of the 45 in the attenuated strain were found to be detected only in samples from one of the strains, and we refer to them as “ON/OFF proteins”. Fold changes of upregulation in the virulent strain ranged from 3.7- to 216.8-fold (Table 1). In the attenuated strain, upregulation ranged from 3.1- to 42.8-fold (Table 2).

### 3.3. Proteins Upregulated in the H. meleagridis Virulent Strain

**Figure 6:**
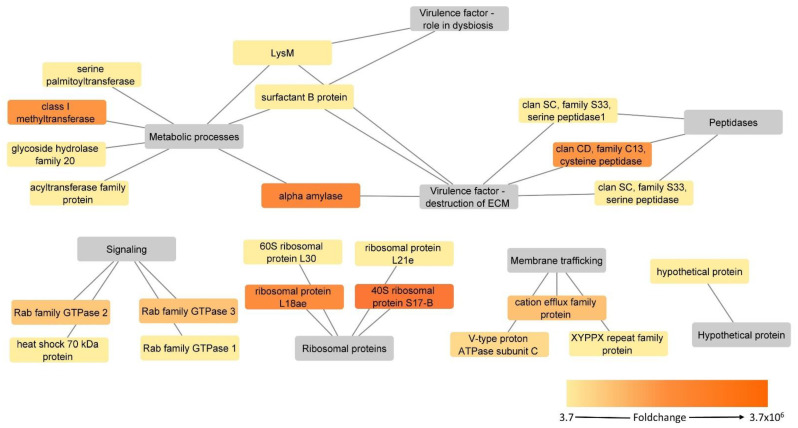
Putative surface-associated, upregulated proteins in the virulent strain of *H. meleagridis*. Protein-function network graphs of *H. meleagridis* proteins with significant upregulation in the virulent strain (>2 fold change and *p* < 0.05). The protein identifications are represented by color-coded source nodes and connected with their proposed functions, represented by the target nodes. The source nodes are color-coded based on each protein fold change upregulation.

Based on their proposed function, the 22 upregulated surface-associated proteins in the virulent strain could be classified into six different categories, them being peptidases, metabolic processes, membrane trafficking, ribosomal proteins, signaling and one hypothetical protein (Figure 6, Table 1).

Two methylesterase-like serine peptidases (Clan SC, family S33) and one asparaginyl endopeptidase-like cysteine peptidase (Clan CD, family C13) were identified as significantly upregulated, with the latter one being an “ON/OFF protein” since it was detected only in the virulent strain. None of the proteins were found to contain a transmembrane domain, but for two of them, a serine peptidase (KAH0796674) and an asparaginyl endopeptidase-like cysteine peptidase (KAH0805360), non-classical secretion was predicted. The other serine peptidase (KAH0803400) was already found significantly upregulated in a previous proteome study [10] (Table 1, Supplementary Table S2).

Seven significantly upregulated proteins were classified as related to metabolic processes, with two of them, class I SAM-dependent methyltransferase and alpha-amylase, being “ON/OFF proteins” (Table 1). For LysM peptidoglycan binding domain-containing protein, a signal peptide was predicted by SignalP server, and three proteins, alpha-amylase, serine palmitoyltransferase and glycoside hydrolase family 20, were identified in analysis with the SecretomeP software for unconventional secretion into the extracellular milieu. Two proteins, alpha-amylase and acyltransferase family protein, were found to possess one or more transmembrane domains. The re-analysis of the proteome data identified LysM and glycoside hydrolase family 20 as significantly upregulated in the virulent proteome [10] (Table 1, Supplementary Table S2). LysM was also found in the exoproteome, together with the surfactant B protein. However, both proteins were not found deregulated in this dataset [12] (Table 1, Supplementary Table S2).

The cation efflux family protein, V-type proton ATPase subunit C and C-domain-containing protein comprise the membrane trafficking group. The first two proteins were also among “ON/OFF proteins” when compared to the attenuated strain. For none of the three proteins neither signal peptide nor non-classical secretion could be predicted, but cation efflux family proteins were shown to contain six transmembrane domains. The same C2 domain-containing protein was also identified as significantly upregulated in the proteome dataset [10] (Table 1, Supplementary Table S2).

Four ribosomal proteins were identified as significantly upregulated in the virulent strain, out of which two of them, 40S ribosomal protein S17-B and ribosomal protein L18ae, were found to be “ON/OFF proteins” as they could not be measured in the attenuated strain (Table 1). For ribosomal protein L21e and 40S ribosomal protein S17-B, non-classical secretion was predicted.

The group of signaling proteins showed some of the overall highest upregulation values. In addition to two “ON/OFF proteins” from the Rab family GTPases, a Ras family GTPase and a heat shock 70kDa protein were identified as being the two proteins with the highest fold change values (Table 1). The heat shock 70kDa protein was predicted to have a signal peptide, whereas one of the Rab family GTPases (KAH0796629) was identified in the analysis for non-classical secretion.

### 3.4. Proteins Upregulated in the H. meleagridis Attenuated Strain

**Figure 7:**
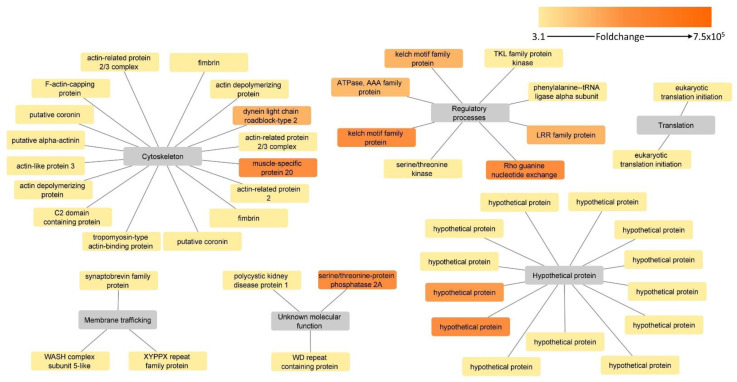
Putative surface-associated, upregulated proteins in the attenuated strain of *H. meleagridis*. Protein-function network graphs of *H. meleagridis* proteins with significant upregulation in the attenuated strain (>2 fold change and *p* < 0.05). The protein identifications are represented by color-coded source nodes and connected with their proposed functions, represented by the target nodes. The source nodes are color-coded based on each protein fold change upregulation.

Upregulated proteins in the attenuated strain were divided into six groups: cytoskeleton, hypothetical proteins, regulatory processes, membrane trafficking, protein translation and unknown molecular function (Figure 7, Table 2).

Cytoskeleton proteins constituted the largest of the above-mentioned groups and were represented by 16 proteins (Table 2). Two proteins within this group, dynein light chain roadblock-type 2 and a muscle-specific protein 20, were found to be “ON/OFF proteins” as they could not be found in the surface-associated fraction of the virulent strain. Interestingly, the re-analysis of proteome data identified muscle-specific protein 20 as upregulated in the attenuated strain, strengthening its predominant presence in the attenuated strain proteome [10] (Table 2, Supplementary Table S2). Only fimbrin was found to possess one transmembrane domain and was also identified in re-analysis of exoproteome data [12] (Table 2, Supplementary Table S2). For three proteins, actin-like protein 3, F-actin capping protein subunit beta and actin-related protein 2/3 complex subunit, non-classical secretion could be predicted.

Thirteen hypothetical proteins were found significantly upregulated in the attenuated strain, with two of them, KAH0806065 and KAH0806186, being “ON/OFF proteins”, as they could not be measured in the virulent strain. None of the upregulated hypothetical proteins contained transmembrane domains, and a signal peptide could not be predicted for any of them. However, two proteins were identified in the analysis with SecretomeP software to be involved in non-classical secretion (Table 2).

The category of regulatory process-related proteins comprised eight proteins, of which the majority (*n* = 5) were “ON/OFF proteins”. None of the proteins contained transmembrane domains, nor were they identified in the analysis with the SignalP software for the presence of signal peptide. However, a protein serine/threonine kinase and a phenylalanine–tRNA ligase were predicted to be secreted by non-classical secretion.

Categories of membrane trafficking/transport, translation and unknown molecular function consisted of proteins for which neither transmembrane domain nor prediction of secretion by either SignalP or SecretomeP software could be identified. However, the majority of them were identified in the re-analysis of the exoproteome data, supporting their association with the cellular surface [12] (Table 2, Supplementary Table S2). Only the HEAT repeat domain-containing protein (KAH0796283) was an “ON/OFF protein” (Table 2).

### 3.5. Confirmation of Differential Gene Expression in Selected Candidates

**Figure 8:**
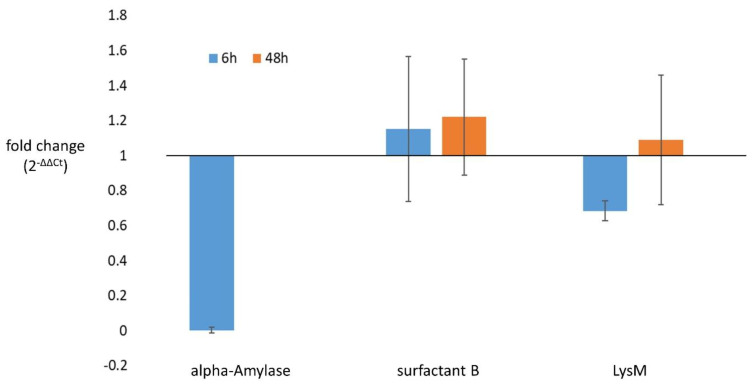
Expression pattern of alpha-amylase, surfactant B and LysM at 6 and 48 h of growth. Gene expression levels for the attenuated and virulent strains were compared at 6 and 48 h of growth. Fold change values were determined with the ΔΔCt method in which the attenuated strain was taken as “treated sample” and virulent as “untreated sample” (ΔΔCT = ΔCT (attenuated strain) − ΔCT (virulent strain), final fold change values were calculated as 2^−∆∆CT^).

Alpha-amylase, Clan CD family C13 asparaginyl endopeptidase-like cysteine pepti-dase, LysM and surfactant B, which were upregulated in the virulent strain, were select-ed for the expression analysis by the RT-qPCR. The alpha-amylase and Clan CD family C13 asparaginyl endopeptidase-like cysteine peptidase were confirmed as “ON/OFF genes”, as no expression could be detected in the attenuated strain after 48 h of growth. In the case of alpha-amylase, some low level of expression was detected in the attenuated strain at 6 h of growth, albeit downregulated when compared to the virulent strain (Figure 8, Supplementary Table S5). The two other genes, LysM and surfactant B, were found to be expressed in both strains at both time points. The LysM showed downregulation in the attenuated strain at 6 h of growth, whereas at 48 h there was almost no difference from the virulent strain (Figure 8, Supplementary Table S5). Surprisingly, the surfactant B transcript showed slight upregulation in the attenuated strain at both time points (Figure 8, Supplementary Table S5). Due to the low number of analyzed samples, statistical analysis could not be performed. The transcriptional regulation of Clan CD, family C13 asparaginyl endopeptidase-like cysteine peptidase and alpha-amylase prompted us to analyze the corresponding genetic loci for the presence of mutations in the attenuated strain; however, no sequence differences between the two strains could be detected (Supplementary File S1,2).

## 4. Discussion

Surfaceome studies provide important information on molecules located on or associated with the cell surface. Due to their location on the cell, such molecules represent the front molecular players in host–parasite interactions [26]. However, current molecular data on *H. meleagridis* lack information on its surface-exposed proteins. Recent proteome studies identified variations between virulent and attenuated *H. meleagridis* strains and recognized potential virulence factors [9,10]. However, as they focused on the analysis of total protein from clarified lysates without any fractionation, the specific identification of proteins located on the cell surface was hindered. The exoproteome study analyzed total protein content in an incubation medium, thereby focusing on extracellular proteins [12]. Even though some surface-exposed proteins that were scraped off the membrane due to experimental conditions were detected within the exoproteome, the cell incubation in a serum-free medium induced stress conditions and the abolition of growth.

In this study, surface-exposed proteins of *H. meleagridis* were tagged with a membrane-impermeable biotin reagent that cannot enter the cell due to its sulfonate group. This method allows biotin labeling of the N-terminal α-amino group of peptides located only on the cell surface and/or outside of the cell. To separate the biotin-bound proteins from the remaining proteome, neutravidin-coated beads were used. This tetrameric protein has a very high affinity for biotin (Ka = 10–15 M) and the lowest nonspecific binding properties among all known biotin-binding proteins [27].

In combination with LC-MS analyses, we quantified a total of 1485 putative surface-associated proteins in both *H. meleagridis* strains. Functional annotation of the identified proteins revealed an overall prominence of structural and metabolic proteins, supporting the hypothesis that surface proteins play an important role in providing structural integrity to the parasite [28].

A high number of ribosomal proteins were found within both samples. This was surprising, as through their association with ribosomes, their location is expected to be cytosolic. However, the same proteins have been found consistently in surface-associated samples from different organisms, which is a strong indicator of their possible association with the cell surface or cell wall or even their secretion into the extracellular medium [29]. In agreement with such a hypothesis, these proteins were reported to possess moonlighting properties in multiple studies, being involved in tumorigenesis, immune signaling and immune development [30]. In *Trichomonas vaginalis*, 23% of the surface-associated proteins identified were ribosomal, and 13% of the proteins in membrane-shed vesicles were identified to be ribosome-related [31,32].

The *H. meleagridis* genome encodes for 11,506 proteins, of which 801 (7%) contain one or more transmembrane domains, 80 (0.7%) contain a signal peptide and 582 (5%) display both [13]. In the present study, only 190 (12.8%) of the surface proteins were identified to have either a transmembrane domain or signal peptide, and 39 (2.6%) of them were identified to have both. Proteins destined to enter the classical secretory system must contain a signal peptide that will result in their translocation to the cell surface [33]. Based on the signal peptides sequence’s conserved nature, bioinformatic analysis can predict whether a protein (i) will enter a classical secretory system, (ii) is part of the cytosolic cell fraction or (iii) will follow an unconventional secretion pathway [34]. In the surface proteome of *H. meleagridis*, 24.4% of proteins were predicted to be unconventionally secreted. This still left a large portion of identified putative surface-associated proteins without any form of tangible connection to the membrane and secretion. This is in agreement with similar studies reporting the surface proteomes of other parasitic protozoa such as *T. vaginalis, Entamoeba histolytica* and *Giardia lamblia*, in which almost half of the identified surface proteins have been found to lack the conventional N-terminal signal peptides or transmembrane domains predicted by bioinformatic analyses [32,35,36]. The mechanisms responsible for unconventional secretion remain an actively researched topic; however, it seems that this process is often triggered as a response to stress, such as starvation, heat shock and even mechanical stress [37].

In our investigations, multiple Rab family proteins were found upregulated in the surface fraction of the virulent strain. Their active role in vesicle formation and vesicular trafficking, analogous to other protozoan parasites, can be hypothesized [38]. Furthermore, the Rab family of small GTPases is known to be involved in pathogenesis-related processes, such as phagocytosis, exocytosis, invasion and evasion of the host immune response [39,40]. These proteins were also found to participate in pinocytosis and the secretion of virulence factors such as the secretion of serine and cysteine proteases in *E. histolytica* [41,42]. It seems that *H. meleagridis* has generally a very prominent vesicle transport given that multiple members of the SNARE families, such as the v-SNARE protein synaptobrevin and t-SNARE protein syntaxin, together with SNARE-complex regulators such as various Rab family GTPases, were identified as surface proteins in both strains [43]. The SNARE machinery plays a crucial role in membrane fusion and in the fusion of vesicles to the plasma membrane [44]. The majority of these proteins from the SNARE family were also identified in the previous proteome and exoproteome studies [10,12]. As for the Rab family GTPases, 16 out of 18 identified in our analysis could also be found in the previous proteome study [10].

In addition to Rab family proteins, several putative virulence factors were found upregulated in the surface fraction of the virulent strain, such as serine and cysteine peptidases, alpha-amylase, LysM peptidoglycan binding domain-containing protein and surfactant B protein.

The cysteine peptidase detected as significantly upregulated in the present study is a Clan CD, family C13, asparaginyl endopeptidase-like cysteine peptidase. In *T. vaginalis,* this protein (referred to as TvLEGU-1) has been classified as a surface protein with high proteolytic activity due to its highly specific range of substrates [45]. Furthermore, it has been suggested that such proteolytic activity can play a major role in the cytoadherence to host cells [46,47]. In the present study, this protein was shown to be one of the “ON/OFF proteins”, as it was detected only in the surface-associated fraction of the virulent strain. This result was supported by RT-qPCR analysis, which demonstrated that the Clan CD, family C13 asparaginyl endopeptidase-like cysteine peptidase gene was not expressed in the attenuated strain. Since transcriptional regulation of this cysteine peptidase could not be linked with any mutation at the corresponding locus in the attenuated strain, it seems that the variation in trans-regulatory elements and/or epigenetic modification between strains is behind the observed phenotype. Taking into account that the cysteine peptidase is solely expressed in the virulent strain, the potentially high relevance of this protein for *Histomonas* in an *in vivo* environment can be hypothesized. Virulent *H. meleagridis* parasites were maintained *in vitro* for just a short period (i.e., 26–28 passages), presumably retaining the bulk expression pattern from *in vivo* conditions. However, after prolonged *in vitro* passaging and occurrence of attenuation, there seems to be no need for this protein. Interestingly, the re-analysis of the proteome and exoproteome LC-MS measurements [10,12] did not detect the Clan CD, family C13 asparaginyl endopeptidase-like cysteine peptidase in neither of the datasets. In contrast to both earlier studies, the present investigation specifically analyzed the surface-exposed proteins of the membrane fraction, suggesting the predominant membrane/surface association of this cysteine peptidase. Considering its cell surface association and exclusive expression in the virulent strain, the role of the Clan CD, family C13 cysteine peptidase in processes involved in the invasion of the host can be hypothesized.

In addition to the cysteine peptidase, two serine peptidases were found to be upregulated in the surface fraction of the virulent strain, with one of them being also detected in higher abundance in the proteome dataset [10], suggesting their general upregulation in the virulent strain. In other organisms, serine peptidases have been reported to be involved in host cell membrane alteration [48,49] and, in the case of other protozoan parasites, to have a proteolytic role in the interaction with host cells [50,51,52]. Therefore, we hypothesize that these two serine peptidases might play a role in assisting with the disruption of the host intestinal epithelium.

The alpha-amylase is another upregulated surface-associated protein that potentially acts as a virulence factor. It was one of the “ON/OFF proteins”, identified only in the virulent dataset of surface-associated proteins. Similarly, to the Clan CD, family C13 asparaginyl endopeptidase-like cysteine peptidase, alpha-amylase was not detected in the re-analysis of the proteome and exoproteome LC-MS measurements [10,12], suggesting its predominant surface association. The sole presence of alpha-amylase in the virulent strain was corroborated by the RT-qPCR analysis since no expression could be detected in the attenuated strain after 48 h of growth. Given the sequence similarities between multiple alpha-amylase genes in the genome, a distinction among them was not possible. Hence, the primer set used to test this protein’s regulation was in fact assessing expression levels of four different (albeit similar) genes. The comparison of genetic loci for all four genes detected no apparent mutation, suggesting a change in trans-regulatory elements and/or variation in epigenetic modification. The alpha-amylase enzyme hydrolyzes alpha bonds of large polysaccharides such as starch that has been a staple addition to the media for optimal growth of *H. meleagridis* and other similar parasites such as *T. vaginalis, E. histolytica* and *G. intestinalis* reviewed in Clark et al., 2002 [53]. Therefore, during *in vitro* cultivation of *H. meleagridis*, alpha-amylase would be one of the enzymes responsible for the hydrolysis of rice starch into glucose. In this context, we observed during *in vitro* cultivation of *H. meleagridis* that the virulent strain consumes the rice starch much better than the attenuated strain (personal observation, data not shown). However, considering that the prolonged cultivation leads to abrogation of alpha-amylase expression, its function is obviously not essential for metabolizing rice starch during *in vitro* growth of *H. meleagridis.* Therefore, the almost exclusively expressed alpha-amylase in the virulent strain, which was cultivated *in vitro* for a short period, points towards its relevance for *in vivo* growth/survival of *H. meleagridis*. In *E. histolytica*, multiple beta-amylases have been reported to allow the protozoan to use the host mucus glycans for its energy metabolism as well as to contribute to the mucosa invasion [54]. An InterProSearch of *H. meleagridis* alpha-amylase revealed the protein to be part of the glycoside hydrolase, family 13, a group of proteins that glycolyze the glycosidic bond between carbohydrates. Analogously to *E. histolytica*, *H. meleagridis* might specifically employ the alpha-amylase’s glycosylic activity to degrade the polysaccharides that form the proteoglycan layer of the extracellular matrix (ECM) into glucose molecules that can be consumed. More so, once the ECM carbohydrate portion is compromised, the aforementioned peptidases, which are upregulated in the virulent strain, will be able to degrade the unprotected protein portion with their endopeptidase activity [55,56]. Ultimately, this might boost the Histomonas virulence and assist with the establishment of infection within the host, similarly to *E. histolytica* that uses both protease and glycosidase activity to disrupt the mucin polymeric network [57,58].

Another protein that adheres to the aforementioned hypothesis is a LysM peptidoglycan binding domain-containing protein. The same LysM domain-containing protein was identified as upregulated in the surface fraction and in the total proteome of the virulent *H. meleagridis* [10], suggesting its general upregulation in the virulent strain. This could not be entirely supported by the RT-qPCR analyses, since although a downregulation of LysM transcripts was detected in the attenuated strain after 6 h of growth, this was not the case after 48 h growth, indicating the regulation of the LysM protein at the translation level. LysM domains are repetitive entities, known to interact with carbohydrates containing N-acetylglucosamine (GlcNAc) moieties, promoting the binding of peptidoglycan in bacteria and chitin in eukaryotes [59]. In *Staphylococcus aureus*, the LysM domain has been shown to mediate the binding of the bacteria to the host’s extracellular membrane proteins [60]. An InterPro Search analysis revealed *Histomonas* LysM-containing protein to possess a glycoside hydrolase 19 domain with chitinase activity. In *E. histolytica*, the same glycosidase activity was hypothesized to play an important role in the disruption of the mucin polymeric network within the caeca [56]. In this respect, we hypothesize that together with the alpha-amylase, the LysM-containing protein of *H. meleagridis* might play a role in binding the protozoan to the ECM of the host, thereby weakening the host epithelial membrane integrity and facilitating the invasion. Considering that *H. meleagridis* survival is dependent on the presence of bacteria, both *in vivo* and *in vitro* [7], the LysM domain-containing protein could also assist in bacterial phagocytosis by the protozoan. This hypothesis is supported by the fact that chickens and turkeys suffering from histomonosis display a severe dysbiosis, presumably influenced by a selective predation of bacteria by the protozoan [61,62].

The surfactant protein B (SP-B) is a further potential virulence factor found upregulated in the surface fraction of the virulent strain, aligning with the aforementioned hypothesis. Since this SP-B protein was not detected as deregulated in LC-MS measurements of both the proteome and exoproteome study [10,12], only a specific upregulation in surface-associated fraction of the virulent strain can be concluded. This observation is supported by RT-qPCR analysis, in which a slight upregulation of the SP-B transcript in the attenuated strain was detected at both time points. SP-B belongs to the saposin-like (SAPLIP) family of proteins, which are predicted to stimulate the lysosomal degradation of several sphingolipids from animals, plants and multiple microorganisms, as reviewed by Zhai et al., 2000 and Bruhn, H. 2005 [63,64]. In *E. histolytica*, surfactant B proteins, defined as amoebopores, are considered to be a major pathogenicity factor for the parasite [65,66], even though it is still unclear whether their activity is on (i) intestinal bacteria, (ii) host cells or (iii) both [67]. In addition to their structural similarities, saposin-like proteins present a similar mode of action. They are mainly involved in the attachment, lysis and fusion of membranes which possess negatively charged phospholipids [68]. Once this protein penetrates the lipid bilayer of a cell, cell death is followed by osmotic lysis [69]. Extrapolating this information to *H. meleagridis*, it can be hypothesized that this SP-B could be an effective virulence factor by its direct action in destroying host intestinal epithelial cells, but also as a player in gut dysbiosis by assisting selective lysis of the intestinal bacteria.

In the attenuated strain of *H. meleagridis,* the most prominent category of upregulated surface-associated proteins is cytoskeleton proteins, representing over one-third of the upregulated proteins in that strain. Comprising actin-related, actin-like and actin-associated proteins (AAPs) such as coronin, fibrin, and alpha-actinin, their action is focused in cytoskeleton remodeling and rearrangements [70]. It has been shown that these proteins are involved in the dynamic remodeling of the actin cytoskeleton, playing a role in multiple physiological processes such as cell migration, endocytosis, cytokinesis and cell morphogenesis [70,71]. In agreement with this, attenuated histomonads demonstrate an amoeboid cellular morphology *in vitro* [72]. Such an amoeboid form provides the parasite with a wider surface area, allowing for a more efficient exchange of nutrients with the surrounding environment [72,73].

Hypothetical proteins (HPs) represent the next big group of upregulated surface-associated proteins in the attenuated strain. A total of 13 HPs with unknown function were found to be more abundantly expressed. Two of them belong to the “ON/OFF proteins” as they were not detected in the virulent strain, suggesting their special importance for the attenuated strain. However, their function still remains to be elucidated.

In conclusion, the present study characterized the surface proteome of *H. meleagridis* and consolidated previous proteomics research conducted on this parasite. Remarkably, many of the identified proteins lack the conventional characteristics common to surface-associated proteins, such as a transmembrane domain or signal peptide. These findings attest to the idea that *H. meleagridis* surface proteome is not static, but rather an intricate system with constant exchanges between plasma and membrane. The virulent strain shows upregulation for multiple virulence factors that are potentially involved in promoting colonization and survival within the host. Furthermore, our analyses show clear signs of *in vitro* adaptation of the attenuated strain. The attenuated strain is overexpressing structural and metabolic proteins that allow the protozoan to thrive in an *in vitro* environment, which confirms our earlier observations with the same cultures [9,10]. We believe our profiling of the *H. meleagridis* surface proteome will facilitate future investigations on the host–parasite interactions and provide a better understanding of its *in vitro* adaptation processes.

## Figures and Tables

**Table 1:** List of upregulated proteins identified with LC-MS analysis on the surface of the virulent *H. meleagridis* strain.

Accession	Protein Name	MW (kDa)	Unique Peptides	Tryptic Peptides	Fold Change	# of TM Domains	Signal-Peptide Prediction	Non-Classical Secretion	Re-Analysis of Proteome LCMS [10]	Re-Analysis of Exoproteome LCMS [12]
**Peptidases**										
KAH0796674	Clan SC, family S33, methylesterase-like serine peptidase	39.3	8	8	4.4	0	No	Secreted	Yes—not upregulated	No
KAH0805360	Clan CD, family C13, asparaginyl endopeptidase-like cysteine peptidase	44.8	2	2	ON/OFF *	0	No	Secreted	-	No
KAH0803400	Clan SC, family S33, methylesterase-like serine peptidase.1	38.9	6	6	7.3	0	No	No	Yes—upregulated (2.12-fold)	No
**Metabolic processes**										
KAH0798244	Class I SAM-dependent methyltransferase	30.4	2	2	ON/OFF *	0	No	No	No	No
KAH0797675	Alpha-amylase, catalytic domain-containing protein	59.6	1	10	ON/OFF *	1	No	Secreted	No	No
KAH0804379	Serine palmitoyltransferase	52.7	2	3	8.6	0	No	Secreted	Yes—not upregulated	No
KAH0804812	LysM peptidoglycan binding domain-containing protein.1	32.2	3	7	3.7	0	Yes	No	Yes—upregulated (5.38-fold)	Yes
KAH0787061	Surfactant B protein	44.1	2	10	9.0	0	No	No	Yes—not upregulated	Yes
KAH0800457	Glycoside hydrolase family 20	12.3	2	5	47.5	0	No	Secreted	Yes—upregulated (35.5-fold)	No
KAH0799877	Acyltransferase family protein	36.2	2	2	7.7	3	No	No	No	No
**Membrane trafficking**										
KAH0804263	Cation efflux family protein	52.5	2	2	ON/OFF *	6	No	No	No	No
KAH0802276	XYPPX repeat family protein/C2 domain-containing protein	32.2	1	2	4.4	0	No	No	Yes—upregulated (3.07-fold)	No
KAH0801182	V-type proton ATPase subunit C	47.3	1	3	ON/OFF *	0	No	No	Yes—not upregulated	No
**Ribosomal proteins**										
KAH0796569	Ribosomal protein L21e.1	18.6	1	5	4.7	0	No	Secreted	Yes—not upregulated	No
KAH0796694	40S ribosomal protein S17-B	14.8	1	7	ON/OFF *	0	No	Secreted	Yes—not upregulated	No
KAH0802566	60S ribosomal protein L30	12.3	1	5	10.7	0	No	No	Yes—not upregulated	No
KAH0798245	Ribosomal protein L18ae	20	1	11	ON/OFF *	0	No	No	No	Yes
**Signaling**										
KAH0796629	Rab family GTPase	19.4	1	2	ON/OFF *	0	No	Secreted	No	No
KAH0806080	Ras family GTPase	21.7	7	7	216.8	0	No	No	Yes—not upregulated	No
KAH0802584	Heat shock 70 kDa protein	72.4	5	6	101.8	0	Yes	No	Yes—not upregulated	Yes
KAH0798120	Rab family GTPase	24.3	1	2	ON/OFF *	0	No	No	No	No
**Hypothetical proteins**										
KAH0799077	Hypothetical protein/Formin	36.9	1	2	4.4	0	No	No	No	No

* Protein detected as a surface-associated protein only in the virulent strain.

**Table 2:** List of upregulated proteins identified with LCMS analysis on the surface of the attenuated *H. meleagridis* strain.

Accession	Protein name	MW (kDa)	Unique Peptides	Tryptic Peptides	Fold Change	# of TM Domains	Signal-Peptide Prediction	Non-Classical Secretion	Re-Analysis of Proteome LCMS [10]	Re-Analysis of Exoproteome LCMS [12]
**Cytoskeleton**										
KAH0806015	Actin-related protein 2	44.5	11	11	3.3	0	No	No	Yes—not upregulated	No
KAH0803799	Fimbrin	68.5	1	16	7.8	1	No	No	No	No
KAH0803847	Actin depolymerizing protein	35.7	5	7	8.6	0	No	No	Yes—not upregulated	No
KAH0804054	Dynein light chain roadblock-type 2	11.2	2	2	ON/OFF *	0	No	No	No	No
KAH0807157	Putative alpha-actinin	130.1	86	86	42.8	0	No	No	Yes—not upregulated	No
KAH0807177	Actin-like protein 3	47.3	2	14	3.4	0	No	Secreted	No	No
KAH0803330	F-actin-capping protein subunit beta	30.6	1	10	6.1	0	No	Secreted	No	No
KAH0801303	Cofilin/tropomyosin-type actin-binding protein	16.1	4	4	5.1	0	No	No	No	No
KAH0800820	C2 domain-containing protein/CH-domain-containing protein	46.6	10	10	3.9	0	No	No	No	No
KAH0799687	Putative coronin	94.8	4	32	21.6	0	No	No	No	No
KAH0806391	Putative coronin	91.3	2	30	8.0	0	No	No	No	No
KAH0799604	Actin depolymerizing protein	35.8	6	8	7.1	0	No	No	No	No
KAH0798726	Muscle-specific protein 20	47.9	3	3	ON/OFF *	0	No	No	No	No
KAH0797693	Fimbrin	70.5	1	16	7.2	0	No	No	No	No
KAH0797549	Actin-related protein 2/3 complex, subunit 1	40	2	17	4.8	0	No	No	No	No
KAH0797350	Actin-related protein 2/3 complex subunit 2	34.3	7	12	3.4	0	No	Secreted	No	No
**Hypothetical proteins**										
KAH0806065	Hypothetical protein.5	14.2	2	2	ON/OFF	0	No	No	No	No
KAH0806131	Hypothetical protein.157	36.9	2	3	8.1	0	No	No	Yes—not upregulated	No
KAH0805781	Hypothetical protein	55.8	3	3	8.3	0	No	No	Yes—not upregulated	No
KAH0805381	Hypothetical protein.62	64.3	11	11	3.8	0	No	No	Yes—not upregulated	No
KAH0804660	Hypothetical protein.92	25.6	6	6	7.2	0	No	No	Yes—not upregulated	No
KAH0807132	Hypothetical protein.68	62.3	9	9	4.8	0	No	No	Yes—not upregulated	No
KAH0802306	Hypothetical protein.128	88.2	3	3	4.6	0	No	No	No	No
KAH0800233	Hypothetical protein.81	23.3	8	8	3.2	0	No	No	No	No
KAH0798642	Hypothetical protein.60	116.8	9	9	3.3	0	No	Secreted	No	No
KAH0806186	Hypothetical protein.3	82.3	2	2	ON/OFF *	0	No	Secreted	No	No
KAH0798386	Hypothetical protein.111	26.3	6	6	4.8	0	No	No	Yes—not upregulated	Yes
KAH0798396	Hypothetical protein.88	41.5	2	9	17.3	0	No	No	Yes—not upregulated	No
KAH0798145	Hypothetical protein.153	91.8	4	4	3.1	0	No	No	Yes—not upregulated	No
**Regulatory processes**										
KAH0796192	Protein serine/threonine kinase, putative	124.9	8	8	10.3	0	No	Secreted	Yes—not upregulated	Yes
KAH0796421	Leucine Rich Repeat family protein	83.9	2	2	ON/OFF *	0	No	No	No	No
KAH0804216	Kelch motif family protein	199.7	2	2	ON/OFF *	0	No	No	Yes—not upregulated	No
KAH0804546	Kelch motif family protein	137.4	4	4	ON/OFF *	0	No	No	Yes—not upregulated	No
KAH0806868	TKL family protein kinase	135.2	2	2	5.8	0	No	No	Yes—not upregulated	No
KAH0802085	Rho guanine nucleotide exchange factor 39	33.6	2	2	ON/OFF *	0	No	No	No	No
KAH0806401	ATPase, AAA family protein	97.5	2	2	ON/OFF *	0	No	No	Yes—not upregulated	No
KAH0798081	Phenylalanine–tRNA ligase alpha subunit	61.3	2	2	25.3	0	No	Secreted	Yes—not upregulated	No
**Membrane trafficking/transport**										
KAH0796205	Synaptobrevin family protein	25	1	4	5.4	0	No	No	Yes—not upregulated	Yes
KAH0802328	WASH complex subunit 5-like	131.5	1	3	3.5	0	No	No	Yes—not upregulated	Yes
KAH0797426	XYPPX repeat family protein	35.8	2	2	11.0	0	No	No	Yes—not upregulated	No
**Translation**										
KAH0796670	Eukaryotic translation initiation factor 3 subunit C isoform X1	80.9	12	14	3.2	0	No	No	Yes—not upregulated	No
KAH0805651	Eukaryotic translation initiation factor 3 subunit 8 N-terminus-domain-containing protein	81.7	9	11	3.2	0	No	No	Yes—not upregulated	Yes
**Unknown molecular function**										
KAH0796283	Serine/threonine-protein phosphatase 2A 65 kDa regulatory subunit A alpha isoform/HEAT repeat family protein	41.4	1	4	ON/OFF *	0	No	No	Yes—not upregulated	Yes
KAH0796931	WD repeat-containing protein 5B isoform X2	39.3	2	2	7.9	0	No	No	No	Yes
KAH0799325	Polycystic kidney disease protein 1-like 3	30.4	2	2	8.9	0	No	No	No	No

* Protein detected as a surface-associated protein only in the attenuated strain.

## Data Availability

The mass spectrometry proteomics data have been deposited to the ProteomeXchange Consortium via the PRIDE [75] partner repository with the dataset identifiers: PXD034844, PXD034834 and PXD034898.

## Supplementary Materials

The following supporting information can be downloaded at: https://www.mdpi.com/article/10.3390/microorganisms10101884/s1. File S1: Nucleic acid alignment of the loci encoding deregulated ClanCD, family C13 asparaginyl endopeptidase-like cysteine peptidase. Complete CDS of GO595_001742 and GPJ56_008847 from the virulent and attenuated strain, respectively, and their 5′-non coding regions were aligned. File S2: Nucleic and amino acid alignments of the loci encoding deregulated alpha-amylase. (a) Nucleic acid alignment of the GO595_009304 and GPJ56_008481 from the virulent and attenuated strain, respectively, and their 5′-non coding regions; (b) Nucleic acid alignment of the GO595_009209 and GPJ56_010552 from the virulent and attenuated strain, respectively, and their 5′-non coding regions; (c) Nucleic acid alignment of the GO595_006104 and GPJ56_010733 from the virulent and attenuated strain, respectively, and their 5′-non coding regions; (d) Nucleic acid alignment of the GO595_005182 and GPJ56_005251 from the virulent and attenuated strain, respectively, and their 5′-non coding regions; (e) amino acid alignment of all deregulated alpha-amylases GO595_009304 (KAH0797675), GO595_009209 (KAH0797990), GO595_006104 (KAH0801069) and GO595_005182 (KAH0802101). Table S1: Primers and probes used in the present study with their respective concentrations and PCR efficiency values [74]. Table S2: List of all *Histomonas meleagridis* surface-associated proteins. Table S3: *Histomonas meleagridis* shotgun proteome re-analysis with *in silico* derived proteome database based on the complete genome. Measurements preformed within study reported in Monoyios et al. 2018 [10] were re-analyzed with new in silico derived proteome database based on the complete genome. Table S4: *Histomonas meleagridis* shotgun exoproteome re-analysis with in silico derived proteome database based on the complete genome. Measurements performed within study reported in Mazumdar et al. 2019 were re-analyzed with new in silico derived proteome database based on the complete genome. Table S5: RT-qPCR data for selected genes, alpha-amylase, Clan CD family C13 asparaginyl endopeptidase-like cysteine peptidase, LysM peptidoglycan binding domain-containing protein and surfactant B, in attenuated and virulent *H. meleagridis*.

## References

[1] Cepicka I., Hampl V., Kulda J. (2010). Critical Taxonomic Revision of Parabasalids with Description of One New Genus and Three New Species. Protist.

[2] Tyzzer E.E. (1920). The flagellate character and reclassification of theparasite producing “blackhead” in turkeys-*Histomonas meleagridis* (Smith). J. Parasitol..

[3] McDougald L.R. (2005). Blackhead Disease (Histomoniasis) in Poultry: A Critical Review. Avian Dis..

[4] Hess M., Liebhart D., Bilic I., Ganas P. (2015). *Histomonas meleagridis*-New Insights into an Old Pathogen. Vet. Parasitol..

[5] Liebhart D., Ganas P., Sulejmanovic T., Hess M. (2017). Histomonosis in Poultry: Previous and Current Strategies for Prevention and Therapy*. Avian Pathol..

[6] Liebhart D., Hess M. (2019). Histomonosis (Blackhead Disease): A Re-Emerging Disease in Turkeys and Chickens. Avian Pathol..

[7] Bilic I., Hess M. (2020). Interplay between *Histomonas meleagridis* and Bacteria: Mutualistic or Predator–Prey?. Trends Parasitol..

[8] Pham A.D.N., Mast J., Magez S., Goddeeris B.M., Carpentier S.C. (2016). The Enrichment of *Histomonas meleagridis* and Its Pathogen-Specific Protein Analysis: A First Step to Shed Light on Its Virulence. Avian Dis..

[9] Monoyios A., Patzl M., Schlosser S., Hess M., Bilic I. (2017). Unravelling the Differences: Comparative Proteomic Analysis of a Clonal Virulent and an Attenuated *Histomonas meleagridis* Strain. Int. J. Parasitol..

[10] Monoyios A., Hummel K., Nöbauer K., Patzl M., Schlosser S., Hess M., Bilic I. (2018). An Alliance of Gel-Based and Gel-Free Proteomic Techniques Displays Substantial Insight Into the Proteome of a Virulent and an Attenuated *Histomonas meleagridis* Strain. Front. Cell. Infect. Microbiol..

[11] Mazumdar R., Endler L., Monoyios A., Hess M., Bilic I. (2017). Establishment of a de Novo Reference Transcriptome of *Histomonas meleagridis* Reveals Basic Insights About Biological Functions and Potential Pathogenic Mechanisms of the Parasite. Protist.

[12] Mazumdar R., Nöbauer K., Hummel K., Hess M., Bilic I. (2019). Molecular Characterization of *Histomonas meleagridis* Exoproteome with Emphasis on Protease Secretion and Parasite-Bacteria Interaction. PLoS ONE.

[13] Palmieri N., Ramires M., Hess M., Bilic I. (2021). Complete Genomes of the Eukaryotic Poultry Parasite *Histomonas meleagridis*: Linking Sequence Analysis with Virulence/Attenuation. BMC Genom..

[14] Holder A.A. (1994). Proteins on the Surface of the Malaria Parasite and Cell Invasion. Parasitology.

[15] Pickering A.C., Fitzgerald J.R. (2020). The Role of Gram-Positive Surface Proteins in Bacterial Niche- and Host-Specialization. Front. Microbiol..

[16] Esbelin J., Santos T., Ribière C., Desvaux M., Viala D., Chambon C., Hébraud M. (2018). Comparison of Three Methods for Cell Surface Proteome Extraction of *Listeria monocytogenes* Biofilms. OMICS J. Integr. Biol..

[17] Hess M., Kolbe T., Grabensteiner E., Prosl H. (2006). Clonal Cultures of *Histomonas meleagridis*, *Tetratrichomonas gallinarum* and a *Blastocystis* sp. Established through Micromanipulation. Parasitology.

[18] Ganas P., Liebhart D., Glösmann M., Hess C., Hess M. (2012). Escherichia Coli Strongly Supports the Growth of *Histomonas meleagridis*, in a Monoxenic Culture, without Influence on Its Pathogenicity. Int. J. Parasitol..

[19] Blum H., Beier H., Gross H.J. (1987). Improved Silver Staining of Plant Proteins, RNA and DNA in Polyacrylamide Gels. Electrophoresis..

[20] Wiśniewski J.R., Zougman A., Nagaraj N., Mann M. (2009). Universal Sample Preparation Method for Proteome Analysis. Nat. Methods.

[21] Wiśniewski J.R. (2016). Quantitative Evaluation of Filter Aided Sample Preparation (FASP) and Multienzyme Digestion FASP Protocols. Anal. Chem..

[22] Gutiérrez A.M., Sotillo J., Schlosser S., Hummel K., Miller I. (2019). Towards Understanding Non-Infectious Growth-Rate Retardation in Growing Pigs. Proteomes.

[23] Zhang X., Smits A.H., Van Tilburg G.B.A., Ovaa H., Huber W., Vermeulen M. (2018). Proteome-Wide Identification of Ubiquitin Interactions Using UbIA-MS. Nat. Protoc..

[24] Livak K.J., Schmittgen T.D. (2001). Analysis of Relative Gene Expression Data Using Real-Time Quantitative PCR and the 2-ΔΔCT Method. Methods.

[25] Bustin S.A., Benes V., Garson J.A., Hellemans J., Huggett J., Kubista M., Mueller R., Nolan T., Pfaffl M.W., Shipley G.L. (2009). The MIQE Guidelines: Minimum Information for Publication of Quantitative Real-Time PCR Experiments. Clin. Chem..

[26] Elschenbroich S., Kim Y., Medin J.A., Kislinger T. (2010). Isolation of Cell Surface Proteins for Mass Spectrometry-Based Proteomics. Expert Rev. Proteom..

[27] Marttila A.T., Laitinen O.H., Airenne K.J., Kulik T., Bayer E.A., Wilchek M., Kulomaa M.S. (2000). Recombinant NeutraLite Avidin: A Non-Glycosylated, Acidic Mutant of Chicken Avidin That Exhibits High Affinity for Biotin and Low Non-Specific Binding Properties. FEBS Lett..

[28] Osborn M.J., Gander J.E., Parisi E., Carson J. (1972). Mechanism of Assembly of the Outer Membrane of *Salmonella Typhimurium*. J. Biol. Chem..

[29] McNamara M., Tzeng S.C., Maier C., Zhang L., Bermudez L.E. (2012). Surface Proteome of “*Mycobacterium avium* subsp. hominissuis” during the Early Stages of Macrophage Infection. Infect. Immun..

[30] Zhou X., Liao W.J., Liao J.M., Liao P., Lu H. (2015). Ribosomal Proteins: Functions beyond the Ribosome. J. Mol. Cell Biol..

[31] Nievas Y.R., Coceres V.M., Midlej V., de Souza W., Benchimol M., Pereira-Neves A., Vashisht A.A., Wohlschlegel J.A., Johnson P.J., de Miguel N. (2018). Membrane-Shed Vesicles from the Parasite *Trichomonas vaginalis*: Characterization and Their Association with Cell Interaction. Cell. Mol. Life Sci..

[32] De Miguel N., Lustig G., Twu O., Chattopadhyay A., Wohlschlegel J.A., Johnson P.J. (2010). Proteome Analysis of the Surface of *Trichomonas vaginalis* Reveals Novel Proteins and Strain-Dependent Differential Expression. Mol. Cell. Proteom..

[33] Delic M., Valli M., Graf A.B., Pfeffer M., Mattanovich D., Gasser B. (2013). The Secretory Pathway: Exploring Yeast Diversity. FEMS Microbiol. Rev..

[34] Zhao L., Poschmann G., Waldera-Lupa D., Rafiee N., Kollmann M., Stühler K. (2019). OutCyte: A Novel Tool for Predicting Unconventional Protein Secretion. Sci. Rep..

[35] Davids B.J., Liu C.M., Hanson E.M., Le C.H.Y., Ang J., Hanevik K., Fischer M., Radunovic M., Langeland N., Ferella M. (2019). Identification of Conserved Candidate Vaccine Antigens in the Surface Proteome of *Giardia lamblia*. Infect. Immun..

[36] Biller L., Matthiesen J., Kühne V., Lotter H., Handal G., Nozaki T., Saito-Nakano Y., Schümann M., Roeder T., Tannich E. (2014). The Cell Surface Proteome of *Entamoeba histolytica*. Mol. Cell. Proteom..

[37] Giuliani F., Grieve A., Rabouille C. (2011). Unconventional Secretion: A Stress on GRASP. Curr. Opin. Cell Biol..

[38] Field M.C., Ali B.R.S., Field H. (1999). GTPases in Protozoan Parasites: Tools for Cell Biology and Chemotherapy. Parasitol. Today.

[39] Zhen Y., Stenmark H. (2015). Cellular Functions of Rab GTPases at a Glance. J. Cell Sci..

[40] Prashar A., Schnettger L., Bernard E.M., Gutierrez M.G. (2017). Rab GTPases in Immunity and Inflammation. Front. Cell. Infect. Microbiol..

[41] Bosch D.E., Siderovski D.P. (2013). G Protein Signaling in the Parasite *Entamoeba histolytica*. Exp. Mol. Med..

[42] Verma K., Srivastava V.K., Datta S. (2020). Rab GTPases Take Centre Stage in Understanding *Entamoeba histolytica* Biology. Small GTPases.

[43] Lorentz A., Baumann A., Vitte J., Blank U. (2012). The SNARE Machinery in Mast Cell Secretion. Front. Immunol..

[44] Gerst J.E. (1999). SNAREs and SNARE Regulators in Membrane Fusion and Exocytosis. Cell. Mol. Life Sci..

[45] Dando P.M., Fortunato M., Smith L., Knight C.G., McKendrick J.E., Barrett A.J. (1999). Pig Kidney Legumain: An Asparaginyl Endopeptidase with Restricted Specificity. Biochem. J..

[46] Arroyo R., Alderete J.F. (1989). *Trichomonas vaginalis* Surface Proteinase Activity Is Necessary for Parasite Adherence to Epithelial Cells. Infect. Immun..

[47] Rendón-Gandarilla F.J., Ramón-Luing L.A., Jaime O.-L., Ivone Rosa d.A., Benchimol M., Arroyo R. (2013). The TvLEGU-1, a Legumain-Like Cysteine Proteinase, Plays a Key Role in *Trichomonas vaginalis* Cytoadherence. Biomed Res. Int..

[48] Kim H.K., Ha Y.R., Yu H.S., Kong H.H., Chung D. (2003). Il Purification and Characterization of a 33 KDa Serine Protease from *Acanthamoeba lugdunensis* KA/E2 Isolated from a Korean Keratitis Patient. Korean J. Parasitol..

[49] Conseil V., Soête M., Dubremetz J.F. (1999). Serine Protease Inhibitors Block Invasion of Host Cells by *Toxoplasma gondii*. Antimicrob. Agents Chemother..

[50] Miller S.A., Binder E.M., Blackman M.J., Carruthers V.B., Kim K. (2001). A Conserved Subtilisin-like Protein TgSUB1 in Microneme Organelles of *Toxoplasma gondii*. J. Biol. Chem..

[51] Withers-Martinez C., Jean L., Blackman M.J. (2004). Subtilisin-like Protease of the Malaria Parasite. Mol. Microbiol..

[52] Hernández-Romano P., Hernández R., Arroyo R., Alderete J.F., López-Villaseñor I. (2010). Identification and Characterization of a Surface-Associated, Subtilisin-like Serine Protease in *Trichomonas vaginalis*. Parasitology.

[53] Clark C.G., Diamond L.S. (2002). Methods for Cultivation of Luminal Parasitic Protists of Clinical Importance. Clin. Microbiol. Rev..

[54] Thibeaux R., Weber C., Hon C.C., Dillies M.A., Avé P., Coppée J.Y., Labruyère E., Guillén N. (2013). Identification of the Virulence Landscape Essential for *Entamoeba histolytica* Invasion of the Human Colon. PLoS Pathog..

[55] Frantz C., Stewart K.M., Weaver V.M. (2010). The Extracellular Matrix at a Glance. J. Cell Sci..

[56] Moncada D., Keller K., Chadee K. (2003). *Entamoeba histolytica* Cysteine Proteinases Disrupt the Polymeric Structure of Colonic Mucin and Alter Its Protective Function. Infect. Immun..

[57] Moncada D., Keller K., Chadee K. (2005). *Entamoeba histolytica*-Secreted Products Degrade Colonic Mucin Oligosaccharides. Infect. Immun..

[58] Tovy A., Hertz R., Siman-Tov R., Syan S., Faust D., Guillen N., Ankri S. (2011). Glucose Starvation Boosts *Entamoeba histolytica* Virulence. PLoS Negl. Trop. Dis..

[59] Mesnage S., Dellarole M., Baxter N.J., Rouget J.B., Dimitrov J.D., Wang N., Fujimoto Y., Hounslow A.M., Lacroix-Desmazes S., Fukase K. (2014). Molecular Basis for Bacterial Peptidoglycan Recognition by LysM Domains. Nat. Commun..

[60] Hirschhausen N., Schlesier T., Peters G., Heilmann C. (2012). Characterization of the Modular Design of the Autolysin/Adhesin Aaa from *Staphylococcus aureus*. PLoS ONE.

[61] Abdelhamid M.K., Quijada N.M., Dzieciol M., Hatfaludi T., Bilic I., Selberherr E., Liebhart D., Hess C., Hess M., Paudel S. (2020). Co-Infection of Chicken Layers With *Histomonas meleagridis* and Avian Pathogenic *Escherichia coli* Is Associated With Dysbiosis, Cecal Colonization and Translocation of the Bacteria From the Gut Lumen. Front. Microbiol..

[62] Abdelhamid M.K., Rychlik I., Hess C., Hatfaludi T., Crhanova M., Karasova D., Lagler J., Liebhart D., Hess M., Paudel S. (2021). Typhlitis Induced by *Histomonas meleagridis* Affects Relative but Not the Absolute *Escherichia coli* Counts and Invasion in the Gut in Turkeys. Vet. Res..

[63] Zhai Y., Saier M.H. (2000). The Amoebapore Superfamily. Biochim. Biophys. Acta Rev. Biomembr..

[64] Bruhn H. (2005). A Short Guided Tour through Functional and Structural Features of Saposin-like Proteins. Biochem. J..

[65] Bujanover S., Katz U., Bracha R., Mirelman D. (2003). A Virulence Attenuated Amoebapore-Less Mutant of *Entamoeba histolytica* and Its Interaction with Host Cells. Int. J. Parasitol..

[66] Bracha R., Nuchamowitz Y., Mirelman D. (2003). Transcriptional Silencing of an Amoebapore Gene in *Entamoeba histolytica*: Molecular Analysis and Effect on Pathogenicity. Eukaryot. Cell.

[67] Ralston K.S., Petri W.A. (2011). Tissue Destruction and Invasion by *Entamoeba histolytica*. Trends Parasitol..

[68] Hawgood S., Derrick M., Poulain F. (1998). Structure and Properties of Surfactant Protein B. Biochim. Biophys. Acta Mol. Basis Dis..

[69] Hirt R.P., de Miguel N., Nakjang S., Dessi D., Liu Y.C., Diaz N., Rappelli P., Acosta-Serrano A., Fiori P.L., Mottram J.C. (2011). *Trichomonas vaginalis* Pathobiology. New Insights from the Genome Sequence. Adv. Parasitol..

[70] Gao J., Nakamura F. (2022). Actin-Associated Proteins and Small Molecules Targeting the Actin Cytoskeleton. Int. J. Mol. Sci..

[71] Lappalainen P. (2016). Actin-Binding Proteins: The Long Road to Understanding the Dynamic Landscape of Cellular Actin Networks. Mol. Biol. Cell.

[72] Gruber J., Ganas P., Hess M. (2017). Long-Term *in Vitro* Cultivation of *Histomonas meleagridis* Coincides with the Dominance of a Very Distinct Phenotype of the Parasite Exhibiting Increased Tenacity and Improved Cell Yields. Parasitology.

[73] Lee D.L., Long P.L., Millard B.J., Bradley J. (1969). The Fine Structure and Method of Feeding of the Tissue Parasitizing Stages of *Histomonas meleagridis*. Parasitology.

[74] Hussain I., Jaskulska B., Hess M., Bilic I. (2015). Detection and Quantification of *Histomonas meleagridis* by Real-Time PCR Targeting Single Copy Genes. Vet. Parasitol..

[75] Perez-Riverol Y., Bai J., Bandla C., García-Seisdedos D., Hewapathirana S., Kamatchinathan S., Kundu D.J., Prakash A., Frericks-Zipper A., Eisenacher M. (2022). The PRIDE Database Resources in 2022: A Hub for Mass Spectrometry-Based Proteomics Evidences. Nucleic Acids Res..

